# *Elisesione*, a new name for *Wesenbergia* Hartman, 1955, and the description of a new species (Annelida, Hesionidae)

**DOI:** 10.3897/zookeys.632.9652

**Published:** 2016-11-16

**Authors:** Sergio I. Salazar-Vallejo

**Affiliations:** 1 El Colegio de la Frontera Sur, Depto. Sistemática y Ecología Acuática, Chetumal, Quintana Roo, México

**Keywords:** Acicular lobe, Hesione, Iceland, polychaetes, simple palps, Wallis and Futuna

## Abstract

*Wesenbergia* Hartman, 1955 (Annelida, Hesionidae) is both preoccupied and a junior homonym of *Wesenbergia* Kryger, 1943 (Hymenoptera, Pteromalidae), and must be renamed. *Elisesione* **nom. n.** is proposed as a replacement name, derived from the combination of the first name of the discoverer, Elise Wesenberg-Lund, and *Hesione* Savigny *in* Lamarck, 1818. *Elisesione mezianei* **sp. n.**, is described from the Wallis and Futuna islands (southwest Pacific). A key to separate *Elisesione mezianei* **sp. n.** from its congener *Elisesione problematica* (Wesenberg-Lund, 1950) is included; further, the record of *Elisesione problematica* for Japan should be regarded as a distinct species because it has palps shorter than antennae (subequal in the type species), and shorter neurochaetal blades (7–9 times longer than wide *vs* 8–12 times longer than wide in the type species).

## Introduction

Hesionid polychaetes are usually colorful polychaetes which are striking because the number of body segments is inversely related to body size. For example, species of *Hesione* Savigny *in* Lamarck, 1818 only have 16 chaetigers during their benthic life, but, are one of the largest representatives in the family with a length of up to 70 mm long in preserved specimens (pers. obs.), although they have been reported to reach 120 mm (Salazar-Vallejo and Rizzo 2009). On the contrary, several genera have smaller species with numerous segments, but they have fragile bodies that break easily; consequently, finding complete specimens is difficult. For example, careful studies have shown that complete specimens with about 30 segments are only 5 mm long (Pleijel et al. 2009).

The phylogenetic affinities among the Hesionidae were assessed by Pleijel (1998). His results indicated two subfamilies (Hesioninae Grube, 1850 and Ophiodrominae Pleijel, 1998), and that Hesioninae includes two tribes: Psamathini Pleijel, 1998 and Hesionini Grube, 1850. Hesionini includes *Hesione*, *Leocrates* Kinberg, 1866, *Leocratides* Ehlers, 1908, *Wesenbergia* Hartman, 1955, and *Dalhousiella* McIntosh, 1901. Pleijel (1998: 114) regarded *Dalhousiella* as *incertae sedis* within Hesionini because he could not study the type specimen, which became lost in the mail. However, *Dalhousiella* is a distinct genus that resembles *Leocratides* because they have biarticulate palps and uniramous parapodia, but they differ because there are no jaws in *Dalhousiella* whereas they are present in *Leocratides*, as indicated elsewhere (McIntosh 1908: 134, 135; Fauvel 1923: 234).

According to Pleijel (1998:107) Hesionini includes species with 21 segments, eight pairs of anterior cirri, bidentate neurochaetae, and pharynx without marginal papillae. The included genera can be separated by the presence of bi-articulated palps [(*Dalhousiella*, *Leocrates*, and *Leocratides*), with biramous (*Leocrates*) or uniramous parapodia (*Dalhousiella* and *Leocratides*), and by the presence of jaws (*Leocratides*), or their absence (*Dalhousiella*)], simple palps (*Wesenbergia*), or the lack of palps (*Hesione*) (Rizzo and Salazar-Vallejo 2014).

Wesenberg-Lund (1950) reported, in one of her many contributions to the Danish Ingolf-Expedition series (Thorson 1969), finding an unusual hesionid polychaete collected in sediments at 550 m depth off Southwest Iceland. The single specimen was damaged but the possession of four appendages on the anterior prostomial margin, separated it from *Hesione* which has only two appendages, and she proposed *Hesionella problematica* as a new genus and new species.

Wesenberg-Lund overlooked a previous publication by Hartman (1939) who had proposed the same genus-group name for another hesionid polychaete, *Hesionella mccullochae*, a small species occurring within the burrows of a lumbrinerid. The homonomy was recognized by Hartman (1955: 41), and she proposed *Wesenbergia* as a replacement name for *Hesionella* Wesenberg-Lund, 1950. Some years later a second replacement name was required for *Hesionella* Friedrich, 1956; Hartmann-Schröder (1959: 74) proposed *Fridericiella* as the replacement, which subsequently became a junior synonym of *Microphthalmus* Mecznikow, 1865 (Westheide (2013), as indicated in WoRMS).

*Wesenbergia* Hartman, 1955 has been recorded for Japan (Imajima 2003) and included in large monographic works (Fauchald 1977, Pleijel 1998), and in keys to hesionid genera (Salazar-Vallejo and Orensanz 2007, Rizzo and Salazar-Vallejo 2014). However, *Wesenbergia* Hartman, 1955 is both preoccupied and a junior homonym of *Wesenbergia* Kryger, 1943, a group of parasitic hymenopterans, and must be replaced. It must be emphasized that detecting such a homonymy could not have been possible even if one had access to the full edition of Neave (1939–1940, *cit.* Evenhuis 2016), but this task is now made easier by consulting the online *Nomenclator Zoologicus* (http://uio.mbl.edu/NomenclatorZoologicus/).

As part of an on-going revision of *Hesione*, materials from several different collections from European, American and Mexican museums or institutions have been examined by the author. In the collections of the Muséum National d'Histoire Naturelle, Paris, a remarkable specimen provided with antennae and simple palps was found, belonging to an undescribed species corresponding to *Wesenbergia*. In this contribution, the new species is described, and because *Wesenbergia* is a junior homonym, a new replacement name is proposed, together with a key to the known species of the genus.

## Material and methods

The holotype was collected during the Musorstom Expedition 7: Wallis and Futuna Islands (Richer de Forges and Menou 1993); it has been deposited in the Muséum National d'Histoire Naturelle, Paris (MNHN). The holotype was photographed with a Canon PowerShot G6 digital camera and a microscope adapter; plates were prepared by compressing a series of photos for each image using Helicon Focus. Immersion of the specimen for 30 sec in an oversaturated methyl-green solution improved the contrast.

## Results

### Hesionidae Grube, 1850
Hesioninae Grube, 1850
Hesionini Grube, 1850

#### Elisesione nomen novum

Taxon classificationAnimaliaPhyllodocidaHesionidae

Hesionella Wesenberg-Lund, 1950: 14.
Wesenbergia Hartman, 1955: 41; Fauchald 1977: 77; Pleijel 1998: 112, 163 (*non*Kryger 1943).

##### Type species.

*Hesionella problematica* Wesenberg-Lund, 1950, by monotypy.

##### Etymology.

The name is a combination of the first name of the late Elise Wesenberg-Lund, and *Hesione*, which is the type genus for the family, but in order to make it more euphonic, the first two letters of the genus-group name are suppressed; the new name emphasizes the similarities between these two genera. Gender feminine.

##### Diagnosis

**(emended).** Hesionini with two antennae; palps simple, lateral to antennae. Eight pairs of tentacular cirri. Dorsal cirri with short or long cirrophores. Notochaetae absent. Aciculae colorless or blackish. Acicular lobes single or double. Neurochaetae with blades bidentate, guards approaching subdistal tooth, or absent. Prepygidial segment with dorsal cirri about 10 times longer than ventral cirri.

##### Remarks.

*Wesenbergia* Kryger, 1943 was proposed for a group of chalcid hymenopterans, but the name was overlooked by Hartman (1955) when she proposed the same genus-group name for hesionid polychaetes. Despite *Wesenbergia* Kryger, 1943 being considered a synonym of *Macromesus* Walker, 1848 within Hymenoptera, the name still cannot be made available (ICZN 1999, Art. 23, Principle of Priority).

Homonymies are not allowed in Zoological Nomenclature (ICZN 1999, Chap. 12) and junior homonyms must be replaced (Art. 60). Further, the Code of Ethics includes (ICZN 1999, Point 3) a recommendation for the procedure, especially if the author(s) involved are alive. There are no junior synonyms available and this explains why a new name must be proposed, and both authors involved are deceased.

As indicated above, *Wesenbergia* Hartman, 1955 is a junior homonym and must be replaced, even though the senior homonym is regarded as a junior synonym (Heqvist 1960). In naming *Wesenbergia*, Hartman used the first word in the compound last name of Elise Wesenberg-Lund. Using this same principle, the new name, *Elisesione*, is derived from the first name of the author.

*Elisesione* nom. n. is closely related to *Hesione* as shown by Ruta et al. (2007). They differ, however, not only by the presence of simple palps in the former, but because the body is more or less cylindrical, not widened medially or posteriorly as in *Hesione* species. In fact, the lateral cushions, which are typically divided into 2-3 sections and can vary on their degree of lateral expansion in *Hesione*, are rather solid, undivided and projected anteriorly in *Elisesione* nom. n. This feature was noted in the original description when the body was characterized as scolopendriform (Wesenberg-Lund 1950: 14). Further, the anterior eyes of *Wesenbergia* (only recorded for the shallow water species), are half-moon shaped and about three times larger than posterior ones; this is another feature not recorded for any *Hesione* species.

Savigny (1822: 39) included four anterior appendages in the generic diagnosis of *Hesione*, but because they were not included in the description (Savigny 1822: 40), nor in the corresponding illustration (his plate 3, figure 3), they were regarded as a mistake. Grube (1867: 65) corrected this and later Chamberlin (1919: 185) used this in his key to genera. However, by regarding *Hesione* as having four antennae and eight pairs of tentacular cirri, de Quatrefages (1866) proposed *Fallacia* for species having two antennae: *Hesione pantherina* Risso, 1826 and *Hesione proctochona* Schmarda, 1861, whereas Claparède (1868: 541) proposed *Telamone* for species having two antennae and six pairs of tentacular cirri with *Hesione sicula* delle Chiaje, 1822 as its only species. *Fallacia* and *Telamone* are junior synonyms of *Hesione* (Fauvel 1911: 374, Chamberlin 1919: 186, Pleijel 1998: 107), and *Hesione sicula* and *Hesione pantherina* have been regarded as synonyms (Fauvel 1923: 233).

##### Distribution.

The two known species in the genus have been found in different ecological conditions and geographical regions. The type species, *Elisesione problematica*, was found in the North Atlantic, off Iceland, in sediments taken at 550 m depth, and the new species, *Elisesione mezianei* sp. n., was collected in the Western South Pacific, in hard substrates in shallow water (35 m), in the Wallis and Futuna Islands. Another species, previously recorded as *Elisesione problematica* from Japan (Imajima 2003) differs from the nominal form in several features. For example, in the Japanese specimens palps are half as long as antennae (rather than about equal-sized), and ventral cirri extend beyond chaetal lobe (rather than short of it); pigmentation also differs because the Japanese specimens are brownish with dorsal cirrostyles banded, whereas the Icelandic specimens are pale yellowish.

##### Key to species of *Elisesione* nomen novum

**Table:** 

1	Acicular lobe single; parapodia with dorsal ceratophores about twice longer than wide; neurochaetal blades with guards	**2**
–	Acicular lobe double; parapodia with dorsal ceratophores 4–5 times longer than wide; neurochaetal blades 1–3 times longer than wide, without guards (palps about 2/3 as long as antennae)	***Elisesione mezianei* sp. n.**
2	Neurochaetal blades 8–12 times longer than wide; palps as long as antennae	***Elisesione problematica* (Wesenberg-Lund, 1950)**
–	Neurochaetal blades 7-9 times longer than wide; palps half as long as antennae	***Elisesione problematicasensu* Imajima, 2003**

#### Elisesione mezianei sp. n.

Taxon classificationAnimaliaPhyllodocidaHesionidae

http://zoobank.org/09A8C65D-DBE4-43AB-9AF7-F3029BF64C7A

Figs 1 , 2

##### Type material.

Holotype (MNHN 1777), Musorstom Expedition 7, Wallis & Futuna Islands, Sta. 536 (12°30.8'S, 176°41'W), Waren Dredge, Waterwitch Bank, 128 km NW off Wallis Island, 27–37 m, coralline rocks, crinoids, crabs, 16 May 1992, B. Richer, coll.

##### Etymology.

This species is named to honor Dr. Tarik Meziane, Curator of Polychaeta in the Muséum National d'Histoire Naturelle, Paris, as an appreciation of his efforts and support to my research activities during many years. The epithet is a noun in apposition.

##### Description.

Holotype (MNHN 1777) complete, subcylindrical, slightly damaged, bent ventrally, many neurochaetal blades broken; 28 mm long, 3 mm wide, 16 chaetigers (right parapodium of chaetiger 7 removed for observation, now kept in plastic vial with holotype).

**Figure 1.**
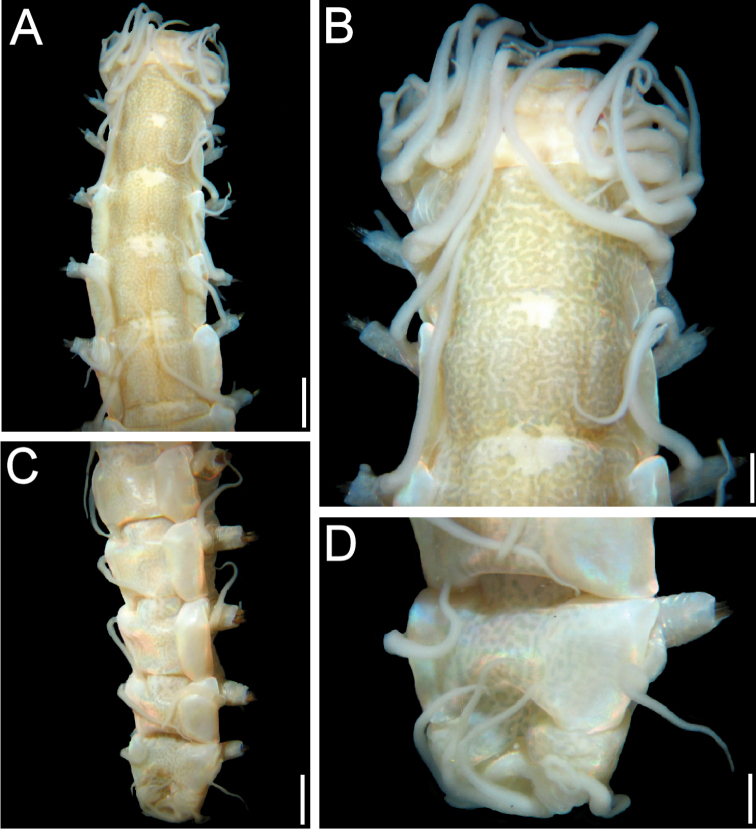
*Elisesione mezianei* sp. n. Holotype (MNHN 1777). **A** Anterior region, dorsal view **B** Anterior end, dorsal view **C** Posterior region, slightly oblique dorsal view **D** Pygidium. Scale bars **A** 1.6 mm, **B** 0.5 mm, **C** 1.2 mm, **D** 0.4 mm.

Body with parallel sides (Fig. 1A), barely tapered posteriorly; pigmentation brownish, with abundant irregular spots variably fused into transverse or longitudinal lines, leaving a mid-dorsal, irregular, wider than long pale area in each segment (Fig. 1B); pigment intensity and definition progressively reduced posteriorly. Lateral and ventral surfaces pale.

**Figure 2.**
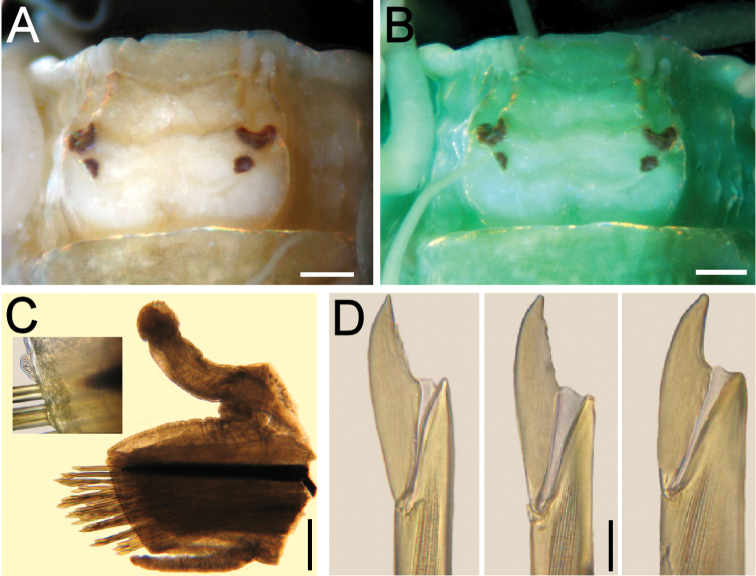
*Elisesione mezianei* sp. n. Holotype (MNHN 1777). **A** Prostomium, dorsal view **B** Same, after methyl-green staining **C** Chaetiger 7, right parapodium, anterior view, dorsal cirrostyle removed, only base left on cirrophore (inset: close-up showing tips of double acicular lobe) **D** Chaetiger 7, neurochaetal blades, variably eroded; the one on the right has a wider handle due to optical interference, not really wider than the others. Scale bars **A, B** 0.27 mm, **C** 0.3 mm, **D** 20 µm.

Prostomium slightly wider than long, anterior margin with a shallow depression, lateral margins rounded, wider medially, posterior margin with a shallow depression, as long as 1/6 prostomial length. Antennae digitate, longer than interocular distance. Palps simple, blunt, 2/3 as long as antennae, positioned at the same level, external to antennae. Eyes blackish, anterior ones half-moon shaped, three times as large as posterior rounded ones (Fig. 2A, B).

Enlarged cirri long, thick, longest one reaches chaetiger 5. Lateral cushions low, projected anteriorly, slightly projected laterally, undivided.

Parapodia with chaetal lobes cylindrical, truncate, longer than wide; dorsal cirri thick with cirrophores cylindrical, 4–5 times longer than wide (Fig. 2C), cirrostyle basally cylindrical, smooth, medially annulated, distally articulated, shorter than body width (without parapodia). Ventral cirri basally smooth, rugose medially and distally, surpassing chaetal lobes.

Acicula black, tapered; acicular lobe double, each lobe blunt, of similar size, barely visible because of chaetal lobe contraction (Fig. 2C, inset). Neurochaetae about 30 per bundle, handle and blade brownish, blade unidentate but some chaetae with subdistal tooth remains, probably eroded; guards not seen (Fig. 2D).

Posterior end tapered into a blunt cone (Fig. 1C); prepygydial segment with asymmetrical cirri, dorsal ones over 10 times longer than ventral ones; pygidium smooth, depressed (Fig. 1D); anus dorso-terminal, open, about 9 anal papillae.

Pharynx not exposed. Oocytes not seen.

##### Remarks.

As indicated in the key above, *Elisesione mezianei* sp. n. differs from both the Icelandic and the Japanese *Elisesione problematica* in parapodial and chaetal features. In *Elisesione mezianei* dorsal ceratophores are long (4–5× longer than wide), the acicular lobe is double, and neurochaetal blades are short (1–3 times longer than wide), whereas in *Elisesione problematica* dorsal ceratophores are short (2× longer than wide), the acicular lobe is single, and neurochaetal blades are long (8–12× longer than wide). Based upon the observation of other similar hesionid specimens, it is clear that these morphological differences are not the result of preservation methods, or prolonged storage in ethanol.

On the contrary, pigmentation patterns can be modified by dissolution in ethanol, because of photo-oxidation, or both, and despite the striking contrast between the two species, they could not be employed as diagnostic features. The pigmentation of *Elisesione mezianei* is long-lasting since it has been in ethanol for at least 16 years, when it was initially sorted-out as part of the Musorstom materials (Salazar-Vallejo 1999). Although they might be regarded of as having a little diagnostic relevance, the dorsal anastomosing thin brownish lines together with the shape and large size of the anterior eyes, are quite remarkable and unique for the genus, and, it must be added, not apparent in any *Hesione* species.

##### Distribution.

*Elisesione mezianei* sp. n. is the second species in a previous monotypic genus and it is apparently rare along its distribution in rocky, shallow water substrates (35 m) in the Southwestern Pacific. The distribution for the genus is rather interesting and difficult to explain. The type species, *Elisesione problematica* (Wesenberg-Lund, 1950) thrives in very cold waters in Iceland, and was also recorded in Japan in sediments at 150-320 m depth (Imajima 2003), whereas the new species, *Elisesione mezianei*, was found in shallow environments in a single locality in the tropical Pacific.

## Discussion

Solving a problem of homonymy in zoological nomenclature is not a remarkable contribution *per se*, especially after 2004 when the *Nomenclator Zoologicus* was available online (Remsen et al. 2006). In fact, during a research visit in Rio de Janeiro, Brazil in 2012, Alexandra Rizzo (Rio de Janeiro State University), and I became aware of this homonymy but decided to wait to gather more information, and especially, to find some means to make more than a mere proposal for a replacement name. In fact, the Wikipedia entry for Hesionidae (https://en.wikipedia.org/wiki/Hesionidae) has an indication that *Wesenbergia* Hartman, 1955 is a junior homonym. The present proposal for a replacement name together with the description of a new species will hopefully be regarded as a better means to solve the problem.

In any case, solving this homonymy problem is by no means a derogatory remark on the impressive publication output of either Elise Wesenberg-Lund or Olga Hartman. They were extremely productive, often published large monographs or revisions, and the former also dealt with a wide variety of invertebrate groups. It was a mistake, a small one, and being related to a formerly monotypic genus, with apparently a single record, this name replacement would not imply a large impact on polychaete taxonomy or benthic ecology, faunal listings or similar efforts.

## Supplementary Material

XML Treatment for Elisesione

XML Treatment for Elisesione mezianei
